# Impact of Trauma Dispatch Algorithm Software on the Rate of Missions of Emergency Medical Services

**DOI:** 10.5812/traumamon.6341

**Published:** 2012-10-10

**Authors:** Reza Alizadeh, Farzad Panahi, Masoud Saghafinia, Keivan Alizadeh, Neusha Barakati, Mohammad Khaje-Daloee

**Affiliations:** 1 Trauma Research Center, Baqiyatallah University of Medical Sciences, Tehran, IR Iran; 2 Department of Ophthalmology, Khatam-al-Anbia Eye Hospital , Faculty of Medicine, Mashhad University of Medical Sciences, Mashhad, IR Iran; 3 Department of Interventional Cardiology, Rajaee Heart Hospital, Tehran University of Medical Sciences, Tehran, IR Iran; 4 Faculty of Medicine, Mashhad University of Medical Sciences, Mashhad, IR Iran; 5 Department of Epidemiology and Statistics, Faculty of Medicine, Mashhad University of Medical Sciences, Mashhad, IR Iran

**Keywords:** Triage, Trauma, Algorithms, Emergency Medical Service, Communication Systems

## Abstract

**Background:**

Trauma still stands atop of the list of emergencies. Transfer of these patients via Emergency Medical Services (EMS) dispatch is critical with regard to importance of timing. This aspect has achieved greater importance due to population increase and telephone triage.

**Objectives:**

We aimed to decrease unnecessary Emergency Medical Services (EMS) missions via a computer program designed for an algorithmic approach for trauma care by nurses involved in EMS, to help them evaluate the case more accurately. We named our program “Trauma Dispatch Algorithm”.

**Materials and Methods:**

First, the most common chief complaints regarding traumatic events were chosen from searching all the calls in December 2008 recorded in Tehran, Iran’s EMS center; and then an algorithm approach was written for them. These algorithms were revised by three traumatologists and emergency medicine specialists, after their approval the algorithms were evaluated by EMS dispatch center for their practicality. Finally all data were turned into computer software. The program was used at the Tehran EMS center; 100 recorded calls assessed with each system were selected randomly. They were evaluated by another traumatologist whether it was necessary to send a team to the site or not.

**Results:**

The age average was 26 years in both groups. The “trauma dispatch algorithm” was significantly effective in reducing the unnecessary missions of EMS by 16% (from 42% to 26%) (P = 0.005).

**Conclusions:**

This program was effective in reducing unnecessary missions. We propose the usage of this system in all EMS centers.

## 1. Background

The increased need for medical care in recent years has resulted in more calls for emergency care; most of these cases can be managed elsewhere (1). It is obvious that when emergency wards are used for non-emergent cases, the expenses would be 2-3 times more than other sectors of a hospital. On the other hand, nurse-to-patient ratio and consequently the quality of service given to patients will decrease. Tele-triage systems seem to be a solution. The topic was introduced in early 90's in the United States when National Health Enhancement System Inc. produced diagnostic algorithms for telephonic triage systems in United States; the system that today covers almost 100 million people nationwide (2). The system is common in other countries as well (3). It is obvious that patients over value their complaints (4) so they should be guided by a nurse (5) who can manage them accurately (6). Such system has not been shaped and used in Iran yet.

## 2. Objectives

This would lead to a decrease in unnecessary Emergency Medical Services (EMS) missions. We think that a computer program designed for an algorithmic approach by nurses, who are involved in EMS, will help them evaluate the case more accurately. We named our program “Trauma Dispatch Algorithm”.

## 3. Materials and Methods

First, all the calls in December 2008 from the Emergency Medical Services (EMS) center in Tehran, Iran were extracted. All these calls were anonymous and patient data remained confidential. From 5979 calls, the most common chief complaints related to motor accidents and traumatic events. They were bone trauma, back and neck trauma, limb trauma, head trauma, chest trauma, bleeding, abdominal pain in adults, abdominal pain in children, low back pain, extremity problems, and joint problems. An algorithm for each chief complaint was formulated (7, 8). These algorithms were designed to help the nurses evaluate whether there is a need to send a team to the accident site or not. If the answer was negative, they told the patients what to do.

These algorithms were evaluated by three traumatologists. The requested changes were made and the final version was reviewed with medical references once more in order to avoid any scientific short-comings. Then they were assessed by another expert panel consisting of Tehran's EMS center personnel and managers to check their practical potential. After approval, all algorithms were given to a software expert and were turned into software compatible with the automation systems currently used in emergency systems nationwide. All nurses were taught how to use the software and algorithms. By using this software, if call duration between dispatching nurse and the caller lasted for more than 100 seconds (which was the mean duration for sending an emergency team with conventional system) a team was expedited independent of the result of the algorithm. We put this limit in order to omit any chance of harming patients due to inexperience of our dispatch nurses using the system and software.

The system was used at the Tehran EMS center for one month. After this period, 100 cases were randomly taken from the recorded calls which were evaluated by the current system and the same amount from the recorded calls which were evaluated by the new system. All the calls were anonymous and patient data remained confidential as well. These recorded calls were evaluated by another traumatologist. To avoid any selection bias, the traumatologist was different from the first panel members and also unaware of the type of triage system. Data were gathered in an SPSS file, version 11 and numeric data were evaluated by student T-test and Non- numeric data were evaluated by Chi-squre test ( P value less than 0.5 was considered significant).

## 4. Results

**Figure 1:**
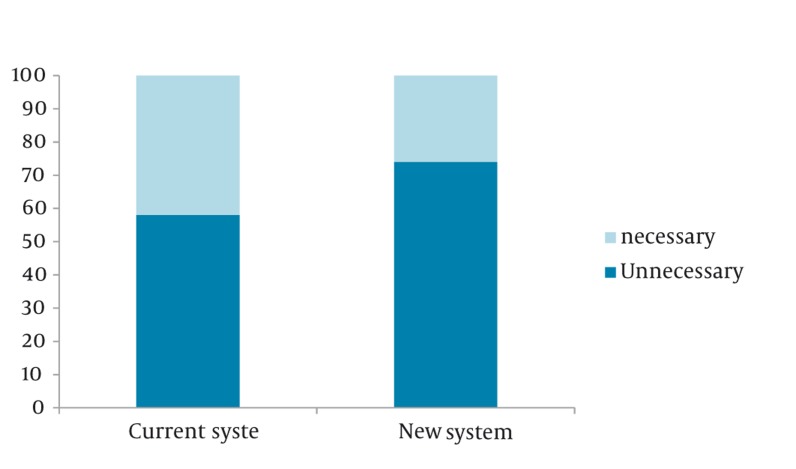
Gender Distribution Between Groups

Two hundred calls were assessed in this study. Mean age of the cases were 26 ± 5 years ( range of 12-36); 80 cases were male (Figure 1). Mean age was 26 ± 4.5 in the control group (range 15 to 38). 78.4% were male in this group. Call duration was 80 ± 10 seconds for the new system and 100±15 for the current system (P < 0.001). No serious or life threatening health problems were observed in all the subjects of our study (Table 1 and 2). Due to the evaluation of the traumatologist, the unnecessary missions had decrease from 42% in control group to 26% in thecase group, which means a 16% decrease (P = 0.005). No call lasted more than 100 seconds, as a result there was no unit mission due to longer than usual emergency call and no call was omitted from the survey.

**Table 1:** Demographic Data of Patients in two Groups

	New system	Current system	*P*
**Male, %**	80	78.4	0.781
**Female, %**	20	21.6	0.834
**Age range, y**	12-36	15-38	-
**Age, y (mean ± SD)**	26 ± 5	26 ± 4.5	0.474
**Emergency Call duration**	80 ± 10	100 ± 15	0.000

**Table 2:** Comparison of the new System vs. Current System Considering the Necessity of Mission

	Current system				New system				*P*
	Necessary		Unnecessary		Necessary		Unnecessary		
**Trauma expert panel decision about emergency mission**	58	58%	42	42%	74	74%	26	26%	0.005

## 5. Discussion

**Figure 2:**
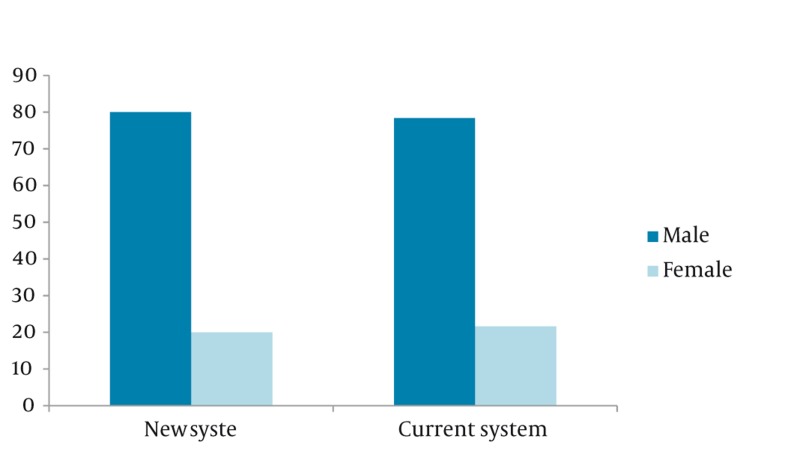
Differences Between 2 Groups in Necessary Missions

The decrease in unnecessary emergency unit dispatch was 16% (from 42% to 26%) (P = 0.005) (Figure 2) which is higher than Hogenbrik report in 2005 (9). Although it is lower than a pilot study in Canada (36 %) (1) and Roth (41 %) (10).

Differences between these results and previous studies mostly arise from the following two factors. First, in some studies both emergent and non-emergent patients have been dealt with. Second, demographic, cultural, social and legal situations vary in different societies. For instance, as a rule in Iran, an emergency unit should be sent for every automobile accident, although obviously this is not necessary in all cases. As a result, unnecessary missions cannot be completely managed.

On the other hand, this new program helped the EMS center nurses to evaluate the cases more rapidly. It is obvious that time is a valuable parameter in emergent cases. This reduction is multiplied by the calls made each month. One of the limitations of this research was the fear of under-triage and penalty for nurses; this may lead to over triage in the current system. Although evaluating the effect of any system on under triage is more important (11), under triage cannot be evaluated truly because of the mentioned limitations.

Furthermore an accurate diagnosis is extremely necessary for assessing the under-triage rate. Diagnosis can be confirmed by two ways: history taking and reviewing medical documents. As long as patients over-express their condition in emergency situations this method is inaccurate. On the other hand gathering medical hospital documents for emergency patients is not feasible, considering the diversity and distribution of medical centers. Other differences between our research and other works arise from differences in EMS facilities, for example helicopter transport which is not a routine route of transportation in Iran (12).

The other fact is that our algorithms are for all people and not specialized for special age ranges e.g. children (13) or elderly patients (14, 15). Considering the reduction made to EMS unnecessary missions, the cost reduction and time consuming decrease is remarkable; use of this new system is recommended in emergency departments.
